# Solitary fibrous tumor of the nasal septum: a case report and literature review

**DOI:** 10.1093/jscr/rjaf556

**Published:** 2025-07-24

**Authors:** Sarah M AlQahtani, Maria R Alabdulaal, Hussain J Aljubran, Ali Almomen

**Affiliations:** Department of Otolaryngology Head and Neck Surgery, King Fahad Specialist Hospital, Dammam 32253, Saudi Arabia; Department of Otolaryngology Head and Neck Surgery, Aljabr Eye and ENT Hospital, Alahsa Health Cluster, Alahsa 36375, Saudi Arabia; Department of Otolaryngology Head and Neck Surgery, Aljabr Eye and ENT Hospital, Alahsa Health Cluster, Alahsa 36375, Saudi Arabia; Department of Otolaryngology Head and Neck Surgery, King Fahad Specialist Hospital, Dammam 32253, Saudi Arabia

**Keywords:** solitary fibrous tumors, endoscopic sinus surgery, nasal tumors, spindle cell neoplasm

## Abstract

Solitary fibrous tumors (SFT) are rare spindle cell neoplasms of mesenchymal origin, typically occurring in the pleura, with extra-pleural manifestations being uncommon. SFTs of the nasal cavity are particularly rare, with limited cases documented in the literature. This report presents the case of a 62-year-old female with a solitary fibrous tumor arising from the nasal septum. The patient presented with a unilateral nasal mass, obstruction, and nasal discharge, which did not improve with antibiotics. The tumor was successfully excised via an endonasal endoscopic approach, and histopathological analysis confirmed the diagnosis of an SFT with positive CD34 and CD99 markers. The patient remained symptom-free with no recurrence over 2 years of follow-up. This case highlights the importance of considering SFTs in the differential diagnosis of nasal masses and underscores the effectiveness of endonasal endoscopic resection as a minimally invasive treatment approach.

## Introduction

Solitary fibrous tumors (SFT) are rare spindle cell neoplasms of mesenchymal origin, commonly found in the pleura [1]. Various extra-pleural sites of SFT have been reported in the literature such as the liver, lung, thyroid, parapharyngeal space, and sublingual glands. SFT on nasal cavities are very rare with few reported cases [2]. Herein, we report a case of solitary fibrous tumor growing from the nasal septum managed successfully by endonasal endoscopic excision underlining its clinical presentation, diagnosis, and management.

## Case report

A 62 years-old female, known for scoliosis, was referred from family medicine to rhinology and skull base clinic for the assessment of unilateral painful swelling of nasal bridge for 2 weeks. Patient reported a history of nasal obstruction, nasal discharge and nasal congestion without improvement after receiving a 7 days course of oral antibiotics. Family history was unremarkable.

**Figure 1:**
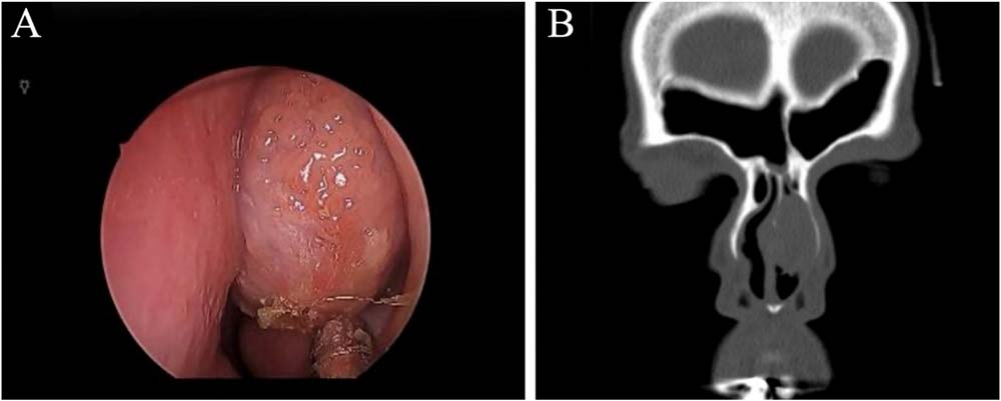
(A) Nasal endoscopic examination showing left nasal septal mass. (B) Computed tomography scan of the paranasal sinuses showing a focal soft tissue lesion arising from the left deviated nasal septum, causing thinning of the left nasal bone measuring 2.2 × 1.7 × 2.7 cm.

A rigid endoscopic examination of the nose showed a unilateral nasal mass originating from the left nasal septum (Fig. 1A). The mass was found above the inferior turbinate and anterior to the middle turbinate, the mass was compressible and bloody on touch. Other otolaryngological examinations were unremarkable, and there were no palpable cervical lymph nodes.

A computed tomography (CT) scan without contrast of the paranasal sinuses showing a focal soft tissue lesion arising from the left deviated nasal septum, causing thinning of the left nasal bone measuring 2.2 × 1.7 × 2.7 cm (Fig. 1B).

**Figure 2:**
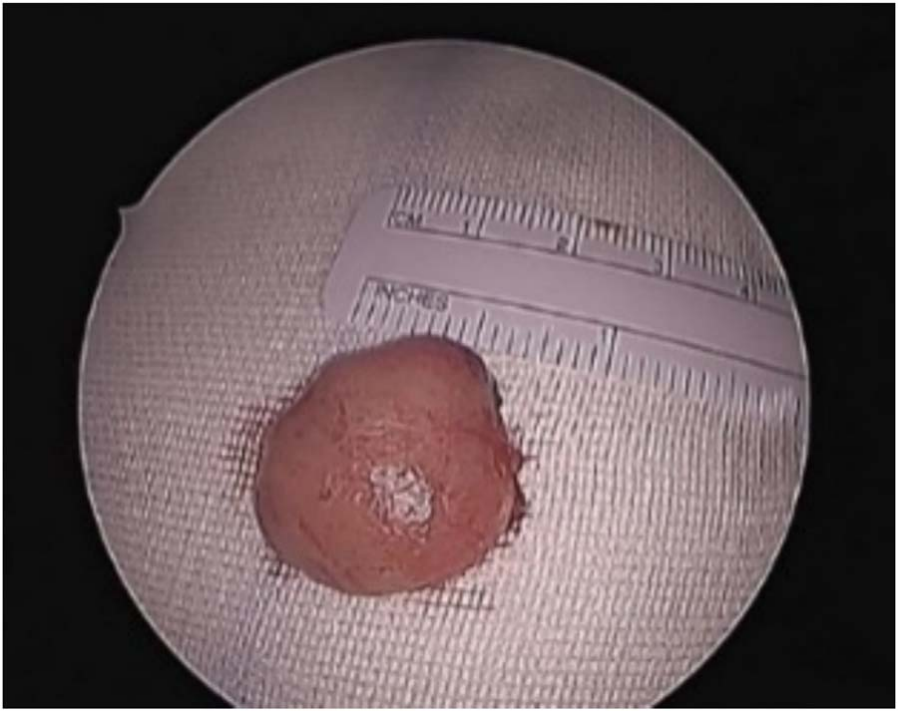
Nasal septal mass post endoscopic excision.

Patient underwent endonasal endoscopic excision of the left nasal septal tumor (Fig. 2). The postoperative recovery of the patient was uneventful. The immunohistochemical examination showed that the tumor cells were positive for CD34 and CD99 while negative for smooth muscle Actin, Desmin, and S100. The final histopathological examination confirmed the diagnosis of a solitary fibrous tumor of the nasal of septum. The patient remained symptoms free with no recurrence on regular follow-ups for 2 years.

## Discussion

SFT are rare tumors first described by Klemperer and Rabin in 1931 in which five cases with primary localization in the pleura were presented [1]. The tumor usually originates from the pleura, but it can also originate from other serosal membranes. With the recent extra-pleural occurrences described in the literature, it is currently assumed that SFT has a mesenchymal origin rather than the mesothelial tissue [3].

SFTs are usually benign neoplasms, with only 10%–15% being malignant [4]. They typically occur in the third to fourth decade of life with a wide age range of presentation from 18 to 79 years and overall, it affects males and females equally [5, 6]. SFTs of head and neck accounting for 5%–27% affecting mostly the orbit and oral cavity [7–9]. The sinonasal tract (SNT) is unusually affected by SFTs, a few SNT SFTs have been described in literature, the majority of which are case studies or brief series [8].

Sinonasal SFTs are usually slow growing tumors presenting as painless mass. Alobid *et al*. reported 21 cases of SNT SFTs. Most common clinical presentations were unilateral mass, rhinorrhea, nasal obstruction, epistaxis, and/or exophthalmos. Anosmia, epiphora, visual disturbance, facial pain and headache can also be present [10]. Our patient presented with painful unilateral mass associated with nasal obstruction and rhinorrhea. As per reported cases in literature, tumor sizes ranged between 1.7 and 8.5 cm [11]. Excised SFTs were described by the authors as well-encapsulated fibrous masses with rich vascularization that were white, reddish or pinkish, oval or round in shape [11].

Imaging examination either CT or Magnetic Resonance Imaging is important during the diagnostic process. On CT SFTs appears on unenhanced CT as a uniform, iso-attenuated mass occupying the nasal cavity and occasionally has internal calcifications. Due to their high vascularity, marked enhancement is typically observed following the administration of contrast material. The nasal septum may deviate depending on tumor size, exhibiting local absorption, remodeling of bone structures, and even reactive sclerosis [12].

A biopsy and pathological examination are necessary in diagnosis. Microscopy, SFTs are composed of spindle cells scattered throughout a collagenous stroma. It is characterized by the presence of hyalinization zones next to collagen deposits. Because of the tumors’ high vascularization, SFT can be confused with hemangio-pericytoma [6, 11]. To confirm the diagnosis of SFTs, immunohistochemical studies are required due to the wide differential diagnosis linked to their histopathologic features. The first-line markers for diagnosing these tumors are bcl-2 and CD34; a tumor that tests negative for both markers is unlikely to be SFT [6, 12]. Unfortunately, CD34 is expressed in several spindle cell neoplasms, including neural tumors and dermatofibrosarcoma protuberans, hence, is not totally specific to SFTs [11]. Furthermore, SFTs are consistently negative for actin, keratin, desmin, and S100 protein and strongly positive for vimentin [12]. In our case, the tumor cells were positive for CD34 and CD99 while negative for smooth muscle Actin, Desmin and S100. These findings supported the diagnosis of SFT.

Complete surgical removal is the mainstay treatment for SFTs. The size, aggressiveness, and extension of the tumor determine the surgical strategy. High risk of local recurrence is associated with tumors larger than 10 cm, positive margins, and the presence of a histologic malignant component [6]. Therefore, the surgical strategy should enable the complete excision of the tumor with clear margins. Different approaches were reported such as lateral rhinotomy, medial maxillectomy, external ethmoidectomy, surgery via the transfacial approach and sphenoidectomy [13, 14].

Endonasal endoscopic resection of SFTs allows good magnification and visualization. However, large and/or bleeding tumors may impair visualization, resulting in incomplete resection [15]. In our case, the endoscopic resection was a suitable approach since the tumor was of small size and the presence of the surgeon’s experience in tackling potential bleeding endoscopically.

## Conclusion

SFTs of nasal cavities are rare neoplasms that pose some challenges to practitioners. It presents with varying clinical and imaging features. Immunohistochemical analysis is crucial to establish the diagnosis as histopathologic features are associated with wide differential diagnosis. The most important prognostic factor in SFTs management is the complete resection of the tumor. The endonasal endoscopic approach is a direct minimally invasive approach with few post-operative complications and a shorter hospital stay.

## Data Availability

All the data that are provided in this study are available upon request from the corresponding author.
